# Innovative Approaches to Advancing Equitable Access to Insomnia Interventions

**DOI:** 10.1007/s40675-026-00385-9

**Published:** 2026-06-23

**Authors:** Sophia Oliver, Kelly Dombek, Anne E. Milner, Allison G. Harvey

**Affiliations:** Department of Psychology, University of California, 2121 Berkeley Way, Berkeley, CA 94720 USA

**Keywords:** Insomnia, Sleep and circadian, Intervention, Dissemination, Equitable access, Transdiagnostic

## Abstract

**Purpose of Review:**

Insomnia disorder is a prevalent and impairing sleep-wake disorder characterized by persistent difficulty initiating or maintaining sleep, accompanied by daytime impairment. Cognitive behavioral therapy for insomnia (CBT-I) is the recommended first-line treatment and has a strong evidence base. Building on this foundation, recent work has increasingly focused on translating these advances into accessible and scalable interventions. This review (1) highlights key challenges in the delivery of insomnia interventions that constrain equitable access and (2) examines emerging approaches that may address these gaps.

**Recent Findings:**

Recent research has identified four primary challenges limiting equitable access to insomnia interventions: high comorbidity with mental health and other sleep and circadian disorders, limited testing and adaptation outside of academic settings, a shortage of trained providers, and structural barriers related to funding and reimbursement. In response, growing evidence supports transdiagnostic treatment approaches, implementation strategies for real-world settings, delivery by non-specialist providers, brief and single-session interventions, and digital and stepped care models as promising pathways to improve access and scalability.

**Summary:**

Our review underscores the importance of moving beyond testing efficacy to address the practical and structural barriers that limit access to evidence-based insomnia interventions. Advancing research that prioritizes real-world application remains essential for narrowing the longstanding gap between evidence and practice and to improve equitable access to effective insomnia interventions.

## Introduction

Insomnia disorder, as defined in the Diagnostic and Statistical Manual of Mental Disorders 5th Edition (DSM-5), is characterized by dissatisfaction with sleep quality or duration, manifesting as difficulty initiating or maintaining sleep at least three nights per week for a minimum of three months, and resulting in clinically significant daytime impairment. It is among the most common sleep-wake disorders, affecting approximately 10–15% of adults as a chronic condition, while up to 33–50% of the population reports at least occasional insomnia symptoms [1–4]. Beyond the high prevalence, insomnia is associated with numerous adverse consequences, including impaired cognitive functioning [5, 6], increased risk for mental and physical health conditions [7], reduced quality of life [8], and substantial economic and healthcare burden [9–11]. Accordingly, insomnia represents a major public health concern, underscoring the need for effective, scalable, and equitable intervention approaches.

Cognitive behavioral therapy for insomnia (CBT-I) is widely recognized as the first-line treatment for insomnia across major clinical guidelines, due to extensive empirical support [12–15]. CBT-I is a multicomponent, non-pharmacological treatment that targets the behavioral and cognitive processes that perpetuate insomnia [16–18]. The behavioral components include sleep restriction [19] and stimulus control [20], while the cognitive components address selective attention to sleep-related threat, discrepancies between subjective and objective sleep, and unhelpful beliefs about sleep [21–23]. There is substantial evidence from randomized controlled trials and meta-analyses demonstrating the efficacy of CBT-I in improving sleep onset latency, wake after sleep onset, sleep efficiency, and daytime functioning across various settings [24–26]. Importantly, CBT-I is also associated with durable treatment gains following the completion of treatment [27, 28].

Building on decades of successful insomnia intervention research, the field is now well positioned for a next phase of scientific inquiry. More specifically, as CBT-I is firmly established as an effective and durable first-line intervention, recent research increasingly shifts our attention toward how evidence-based insomnia interventions can achieve broader real-world impact. This shift reflects longstanding evidence that behavioral sleep medicine services remain insufficient relative to population need, with research documenting a limited number of clinicians trained in CBT-I and uneven availability across care settings [29–31]. Research examining routine clinical delivery further shows that behavioral treatments for insomnia are underutilized, with many individuals receiving pharmacological management or no treatment despite guideline recommendations [32, 33]. In addition, this work highlights structural barriers, including referral pathways, provider capacity, and resource constraints, that shape whether those most affected by this highly prevalent disorder are actually able to access effective care [29, 30, 32].

These patterns have become a central theme in recent review work synthesizing the field’s progress and outlining priorities for extending the impact of CBT-I [34–37]. For example, Muench et al. [37] explicitly ask, “What’s next?”, reflecting the growing consensus that the public health impact of CBT-I depends not only on treatment effectiveness but on how interventions can be delivered across healthcare systems, clinical settings, and populations with varying levels of clinical complexity and resource availability. Importantly, these access constraints are not uniformly distributed across populations or care settings, underscoring the need to consider equitable access as a central dimension of insomnia intervention research [36, 37]. These reviews emphasize that improving population-level impact requires innovation not only in treatment content, but in the systems and structures through which care is delivered.

Taken together, this body of work suggests that insomnia intervention research is entering a phase that builds on decades of successful treatment development and increasingly focuses on translating these advances into broader impact. This shift reflects the widely recognized research-to-practice gap across healthcare, where evidence-based practices often require substantial time to achieve widespread clinical adoption, with estimates suggesting an average lag of approximately 17 years [38–40]. Accordingly, the goal of this review is to build on this momentum by synthesizing four key challenges that limit access to insomnia interventions and highlighting emerging opportunities that may support more effective, scalable, and equitable delivery.

## Challenges and Opportunities for Equitable Access to Insomnia Interventions

### Challenge 1: Insomnia Often Co-Occurs with Other Conditions

In community samples, insomnia frequently co-occurs with other sleep and circadian rhythm disturbances, including delayed sleep-wake phase disorder, irregular bed and wake times, obstructive sleep apnea, and related conditions [41, 42]. Furthermore, people experiencing various mental health conditions commonly report multiple sleep and circadian problems, including but beyond insomnia [43–45]. For example, people diagnosed with depression may experience insomnia alongside hypersomnia, delayed phase disorder, and irregular bed and wake times, reflecting broader sleep-wake dysregulation [46]. These comorbid patterns have important implications for intervention; because standard CBT-I protocols are designed to specifically target insomnia symptoms, they may not adequately address the broader range of difficulties that characterize real-world clinical presentations. In other words, there may be a mismatch between the comorbidity and complexity of the problems patients face and the treatments available [47].

### Opportunity 1: Transdiagnostic Approaches

One opportunity that arises from the observation that insomnia often co-occurs with other conditions is to consider taking a transdiagnostic approach. This involves focusing on the mechanistic processes that are in common across sleep and circadian disorders, rather than treating a single sleep or circadian diagnosis in isolation. This suggestion arises because treatment studies on the sleep problems that people diagnosed with mental disorders often experience tend to be disorder-focused. That is, they treat a specific “single-disorder” sleep or circadian problem (e.g., insomnia) in a specific population (e.g., bipolar disorder). However, this approach neglects the high rates of comorbidity in the sleep and circadian problems that are experienced. For example, in one study, fewer than 10% of participants diagnosed with a mental disorder met criteria for only one sleep or circadian problem. Additionally, more than 80% of people met criteria for at least one subdiagnostic sleep or circadian problem [41]. These data raise the possibility that focusing on insomnia with CBT-I may not fully meet the needs of many people.

As an attempt to address this issue, we developed the Transdiagnostic Sleep and Circadian Intervention or TSC [47]. TSC is designed to provide one short, scalable treatment protocol to target a range of sleep and circadian maintenance processes across a range of mental disorders. The hope is that this transdiagnostic solution will be efficient in time and cost as (a) clinicians can be trained in one protocol that is helpful across sleep and circadian problems and across mental disorders and (b) patients can receive one protocol that would help the comorbid sleep and circadian problems they are experiencing. TSC is designed to address and promote improvement along the six dimensions that comprise the Sleep Health Framework [48] as these are the basic building blocks of sleep health in common across sleep and circadian problems. TSC includes CBT-I, but addresses a much broader range of sleep and circadian dysfunction (e.g., advanced phase, nightmares, adherence to CPAP, sleep in difficult environments) experienced by people diagnosed with a wide range of mental disorders (e.g., bipolar disorder, schizophrenia, depression, anxiety).

TSC is typically delivered in eight 50-minute sessions, however, shorter versions have been tested. TSC is grounded in basic sleep and circadian science and draws principles from four existing evidence-based treatments including CBT-I, Interpersonal and Social Rhythm Therapy [49], Chronotherapy [50, 51], and Motivational Interviewing [52]. TSC takes a modular approach based on evidence that a modular treatment has better uptake by clinicians in routine practice settings [53]. Also, within a modular approach, treatment sessions can be ‘personalized’ to the specific sleep and circadian problem(s) experienced by each patient and thus may be more time efficient. TSC typically includes four cross-cutting modules featured in every session (case formulation, education, motivational enhancement, and goal-setting), four core modules that constitute the basic building blocks of sleep health and apply to the vast majority of people (establishing regular sleep-wake times including learning a wind-down and wake-up routine, improving daytime functioning, correcting unhelpful sleep-related beliefs, and maintaining behavior change), and seven optional modules used less commonly, depending on the needs of each person (improving sleep efficiency, reducing time in bed, addressing delayed or advanced phase through appropriately timed light exposure and gradual behavioral adjustments to sleep-wake timing, reducing sleep-related worry/vigilance, promoting compliance with CPAP/exposure therapy for claustrophobic reactions to CPAP, negotiating sleep in a complicated environment, and treating nightmares). However, modules can be shifted from optional to core and modules can be added to meet the needs of specific groups of people. Evidence for TSC continues to accrue, including in adolescents [54], patients in medical settings [55, 56] and adults who met diagnostic criteria for mental disorder diagnoses [41, 57, 58]. Importantly, there is evidence that TSC can be successfully delivered by community mental health providers who received a day or less of training and minimal supervision (due to the resource constraints of the context) [57]. The results were also encouraging when the community providers were trained by other community providers (i.e., when they were not trained by the “expert” university-based providers) [59]. Taken together, transdiagnostic interventions such as TSC may offer a practical and scalable approach for addressing the complex, comorbid sleep and circadian difficulties that characterize real-world clinical populations and extend beyond insomnia symptoms alone.

### Challenge 2: Insufficient Testing and Adaptation in Nonacademic Settings

Much of the evidence base for insomnia interventions has been generated within university clinics and academic medical centers. Thus, there is a great need for testing and possibly adaptation in routine practice settings [60, 61]. This is important because the existing research may not fully represent the constraints that shape routine care, and relatively few studies have evaluated insomnia interventions in real-world settings. Addressing this gap will require strong efforts to evaluate and, depending on the results, potentially adapt behavioral sleep interventions to nonacademic settings, ensuring they remain effective and are feasible to implement in contexts where most patients receive care.

### Opportunity 2: Dissemination and Implementation

Dissemination of evidence-based insomnia treatments requires attention both to the strategies used to support implementation and the contexts in which interventions are delivered. Even effective treatments, such as CBT-I or TSC, do not scale automatically, and structured dissemination efforts are necessary to ensure sustainable delivery across diverse settings. Active implementation strategies, particularly facilitation, are central to scaling insomnia treatments. Facilitation involves guiding clinicians and organizations through adoption, integration, and ongoing adaptation of evidence-based treatments [62]. In community mental health centers (CMHCs), facilitation has enabled providers to deliver TSC effectively after only brief training, and with a focus on treatment fit to organizational constraints [57, 59]. Similarly, the Veterans Health Administration (VA) has successfully disseminated CBT-I through structured training, consultation, and system-level support, showing improvements in clinician competence, patient outcomes, and sustained program delivery [63–65].

Implementation frameworks such as i-PARIHS (Integrated Promoting Action on Research Implementation in Health Service) and RE-AIM (Reach, Effectiveness, Adoption, Implementation, and Maintenance) can guide these dissemination efforts systematically. I-PARIHS emphasizes the interplay of context, evidence, and facilitation, which provides a roadmap for tailoring interventions to organizational capacity [66–68]. RE-AIM provides a complementary evaluation lens, assessing not only efficacy but reach, provider adoption, fidelity, and long-term sustainment. Applying these frameworks supports the use of evidence-based strategies for implementing interventions in ways that fit local contexts and optimize outcomes.

Contextual adaptation is sometimes needed when translating insomnia interventions from academic research to community-based settings. For example, the Sleep Well! program adapted behavioral sleep interventions for urban primary care by integrating brief behavioral modules and caregiver-focused education to improve uptake and relevance [69]. In inpatient psychiatric wards, transdiagnostic interventions have been adapted to account for high patient acuity, complex comorbidities, and shortened treatment windows, while preserving core therapeutic components [70].

Adapting interventions to the cultural context is especially important when serving racial and ethnically minoritized communities or trauma-affected populations, including refugees and asylum seekers. Dumser et al. [71] developed the STARS group intervention, a down-scaled, culturally adapted protocol that drew on three main sources of evidence-based treatments: CBT-I [16–18, 72], Image Rehearsal Therapy for trauma-affected refugees [73], and TSC [47]. Similarly, early pilot work adapting digital sleep intervention for refugee populations suggests that culturally sensitive and context-appropriate modifications can enhance engagement and outcomes, even with limited resources [74].

Several structural and individual barriers impede large-scale adoption. Inadequate clinician time and competing organizational priorities constrain implementation [75, 76]. Provider-level barriers include inadequate training and knowledge about behavioral sleep treatments and limited screening and referral practices, while patient-level barriers include logistical challenges in accessing care [77]. Framework-guided implementation can help address these multi-level barriers by assessing resources and recipient needs.

### Challenge 3: Shortage of Trained Providers

Access to effective insomnia interventions is further limited by the insufficient number of providers trained in CBT-I [29–31, 36]. Indeed, behavioral sleep medicine training opportunities remain limited, and many clinicians report barriers such as time constraints, lack of institutional support, or perceived complexity of treatment protocols. This shortage of therapists substantially restricts access to evidence-based insomnia care, particularly for individuals in under-resourced settings where specialty sleep services are often scarce or nonexistent [78]. As a result, even individuals who are aware of effective interventions may be unable to receive them, exacerbating disparities in access. Expanding access will likely require innovative dissemination strategies, including training non-specialist providers or integrating brief, evidence-based insomnia interventions into primary care and community mental health settings.

### Opportunity 3: Non-Specialists and Brief or Single-Session Interventions

To address the insufficient number of trained providers, training non-specialist providers (e.g., primary care clinicians, nurses, allied health professionals) has emerged as a feasible solution, particularly when paired with structured facilitation and scalable intervention protocols [57, 59, 79–81]. These approaches are often supported by modular intervention designs that preserve core treatment elements to maintain fidelity while allowing optional components to be adapted to patient needs or contextual constraints. Encouragingly, community providers have demonstrated the ability to deliver evidence-based insomnia interventions in both community mental health settings [57] and culturally adapted interventions for refugee populations [71].

Single-session interventions also offer a promising and scalable approach to increasing access to care while reducing provider burden [82]. These interventions typically consist of a single in-person or virtual visit with a program or provider focused on a key component of an empirically supported treatment [83]. By substantially reducing the treatment length, single-session interventions have the potential to reach larger populations while lowering barriers related to time, cost, and provider availability. Importantly, briefer formats may also improve treatment adherence, as high dropout rates are often observed in longer treatment protocols [7, 84].

A growing body of evidence supports the effectiveness of single-session interventions across a range of mental health concerns [85, 86]. Several studies have begun exploring whether key components of CBT-I can be delivered effectively in brief formats. For instance, in an RCT, Edinger et al. [12] compared varying numbers of CBT-I sessions and found that while four sessions optimized outcomes, a single session of CBT-I performed better than both two- and eight-session lengths. These findings highlight the possibility that treatment length does not necessarily correspond to greater therapeutic benefit and that clinically meaningful improvements can be achieved even with very brief interventions.

Subsequent work has continued to build on these early findings. Ellis et al. [87] conducted an RCT evaluating a “one-shot” CBT-I designed to treat acute insomnia, consisting of a self-help pamphlet paired with a single 60 to 70-minute therapy session. At one-month follow-up, the remission rate was 60% in the intervention group compared to only 15% in the waitlist-control. Similarly, Amra et al. [88] found that a brief group-based CBT-I session following two weeks of sleep-diary monitoring reduced insomnia severity among healthcare workers experiencing acute insomnia. Additional studies further suggest that single-session formats may be beneficial across diverse clinical populations and delivery contexts. For example, Boullin et al. [89] demonstrated improvements in sleep outcomes following a single-session CBT-I intervention for acute insomnia delivered in both group and individual formats. In other clinical contexts, Chevalier et al. [90] reported improvements in sleep outcomes with high patient acceptability following a single-session educational workshop for cancer survivors with insomnia, while Okun and Glidewell [91] observed reductions in insomnia symptoms following a four-hour CBT-I workshop.

Importantly, single-session interventions are not intended to replace longer, multi-session treatments but rather to extend the reach of evidence-based care. While briefer interventions naturally provide fewer opportunities for ongoing skills practice and personalization compared to traditional multi-session protocols, they may serve as an important first step in care or a lower-intensity option for individuals with emerging or less complex sleep difficulties [92, 93]. Encouragingly, early work examining longer-term outcomes suggests that meaningful improvements can persist beyond the initial intervention [94]. As such, brief and single-session interventions present a highly promising and scalable pathway toward increasing equity and accessibility of insomnia interventions, while remaining effective.

### Challenge 4: Funding and Reimbursement Barriers

Internationally, chronic insomnia is associated with significant economic losses [95, 96] due to higher healthcare utilization and indirect costs including work absenteeism, disability, and markedly reduced quality of life [10, 97–99]. However, healthcare financing structures do not consistently support the delivery of behavioral sleep medicine services. Behavioral treatments, such as CBT-I, are often inadequately reimbursed relative to pharmacological options, despite evidence demonstrating their superior long-term efficacy and safety [30, 100].

These funding structures contribute to a mismatch between clinical guidelines recommending CBT-I and the resources available to support its delivery in routine care. Take the example of the healthcare system in the United States, where low reimbursement rates for behavioral health services can increase out-of-pocket costs for patients while reducing incentives for clinics and individual providers to adopt or prioritize behavioral insomnia interventions [78]. These financial barriers may be particularly pronounced in under-resourced or community-based settings where limited budgets and competing priorities can favor shorter-term treatment approaches over behavioral interventions that require more time or specialized training. Implementation work examining efforts to expand access to CBT-I suggests that improving access requires coordinated efforts across policy, funding mechanisms, and health-system infrastructure to support the sustainable delivery of behavioral sleep interventions [101].

### Opportunity 4: Digital and Stepped Care Models

In response to this mismatch between burden and investment, the American Academy of Sleep Medicine has recommended leveraging scalable digital treatment approaches to expand access to evidence-based care [102]. Digital treatment options already show promising signs for increasing accessibility. Studies of the Sleepio platform, a 6-week digital CBT (dCBT) treatment for insomnia, project that national adoption of this treatment could reduce primary care costs by £20 million in the UK [103], and Sleepio was also found to be more cost-effective than pharmacotherapy, individual CBT, and group CBT [104]. Sleepio has been implemented within the UK National Health Service (NHS), including large-scale regional rollouts in which it has been made freely available to patients, and is also offered through some insurance plans in the United States [103]. Similarly, studies of Somryst (formerly SHUTi), the first FDA-approved digital treatment for insomnia, show reductions in healthcare costs by over $1000 per year per patient [105]. The use of digital sleep health tools has emerged as a partial solution to the treatment gap caused by underfunding in large healthcare systems.

Despite their promise, challenges to implementing digital interventions include engagement, adherence, and usability. Many patients report a preference for therapist-led CBT‑I, describing digital programs as lacking human connection and adequate personalization [106]. Developers are addressing these concerns by incorporating easy-to-use and engaging graphical user interfaces, automated reminders, and adaptive treatment algorithms, which increase treatment uptake and satisfaction [107]. Interventions tailored to specific comorbidities also show promise [108], although transdiagnostic treatment research suggests that proliferation in disorder-specific protocols may not be necessary [109, 110]. Instead, digital interventions grounded in a transdiagnostic framework may offer a more efficient and scalable solution by targeting shared maintenance mechanisms across comorbid sleep and circadian problems.

Digital interventions can also be integrated into stepped-care models designed to deliver insomnia care efficiently across healthcare systems. In stepped-care approaches, patients begin with the least resource-intensive intervention likely to be effective, such as self-help materials or digital CBT-I, and are “stepped up” to more intensive treatments if symptoms persist. Espie [111] originally proposed stepped care as a population-level strategy for increasing access to CBT-I by organizing treatment into a hierarchy of progressively more intensive interventions, while preserving core behavioral treatment principles across levels.

Empirical work has begun to evaluate these models in clinical settings. For example, Vincent and Walsh [112] evaluated the implementation of a stepped-care insomnia program in routine clinical practice in which patients first received low-intensity interventions and were subsequently offered therapist-delivered treatment if symptoms did not sufficiently improve. Their findings suggested that a substantial proportion of patients experienced meaningful improvements with lower-intensity treatments alone, allowing specialist behavioral sleep treatment resources to be reserved for individuals requiring more intensive care [112]. Another trial conducted in primary care settings demonstrated that integrating digital and therapist-delivered CBT-I within a stepped model was feasible and associated with improvements in insomnia outcomes [113]. In addition, the RESTING study protocol outlines an ongoing RCT evaluating stepped-care CBT-I in general practice [114]. By matching treatment intensity to patient need, stepped-care approaches may improve efficiency while maintaining treatment effectiveness through more strategic allocation of clinical resources [115]. Emerging machine-learning approaches may further enhance these models by predicting treatment outcomes and assigning patients to the most optimal level of care [116, 117].

Together, these innovations suggest that digital insomnia treatments and stepped care models could substantially narrow the treatment gap and better align healthcare investment with the true burden of chronic insomnia.

## Conclusions

Insomnia remains a highly prevalent and consequential disorder, yet access to effective evidence-based treatments continues to lag far behind need. While this review is far from exhaustive, we seek to highlight a critical point; namely, meaningful progress is being made to bridge the persistent gap between research and practice for insomnia care. Across diverse populations and settings, researchers and clinicians are actively developing, testing, and implementing strategies to expand the reach, adoption, and sustainability of evidence-based insomnia interventions. The work highlighted in this review shows that real progress in insomnia treatment is underway. Transdiagnostic approaches are expanding the focus beyond single-disorder insomnia, addressing comorbid conditions with a single protocol. Dissemination efforts, guided by frameworks such as iPARIHS and RE-AIM, are helping sleep interventions reach community mental health centers, primary care, and other under-resourced settings, often with promising outcomes even when delivered by non-specialist providers. Brief and single-session interventions are lowering barriers related to provider shortages, demonstrating that meaningful improvements can be achieved with scalable, time-efficient strategies. Finally, digital and stepped care models are leveraging technology to broader access, optimizing treatment intensity, and reducing costs, making evidence-based insomnia care more feasible for healthcare systems and patients alike.

Looking forward, continued progress will depend on further refining interventions to meet the needs of diverse populations, integrating innovative delivery strategies, and rigorously evaluating implementation efforts in community-based settings. Priorities include expanding the reach of scalable approaches, ensuring equitable access across communities, and sustaining long-term treatment effects. By building on these emerging pathways, the field is shaping a future where effective insomnia care is equitably accessible to those who need it most.

## Key References

- Morin CM, Bootzin RR, Buysse DJ, Edinger JD, Espie CA, Lichstein KL. Psychological and behavioral treatment of insomnia:update of the recent evidence (1998-2004). Sleep. 2006;29:1398–414.10.1093/sleep/29.11.1398.
  - This study demonstrates that CBT-I produces durable treatment effects, supporting its long-term clinical utility.
- Trauer JM, Qian MY, Doyle JS, Rajaratnam SMW, Cunnington D. Cognitive Behavioral Therapy for Chronic Insomnia: A Systematic Review and Meta-analysis. Ann Intern Med. 2015;163:191–204.10.7326/M14-2841.
  - This meta-analysis demonstrates that CBT-I is effective across various sleep outcomes.
- Edinger JD, Arnedt JT, Bertisch SM, Carney CE, Harrington JJ, Lichstein KL, et al. Behavioral and psychological treatments for chronic insomnia disorder in adults: an American Academy of Sleep Medicine clinical practice guideline. J Clin Sleep Med JCSM Off Publ Am Acad Sleep Med. 2021;17:255–62. 10.5664/jcsm.8986.
  - This clinical guideline establishes CBT-I as the first-line treatment for chronic insomnia in adults.
- Koffel E, Bramoweth AD, Ulmer CS. Increasing access to and utilization of cognitive behavioral therapy for insomnia (CBT-I): a narrative review. J Gen Intern Med. 2018;33:955–62.10.1007/s11606-018-4390-1.
  - This review highlights the gap between CBT-I efficacy and real-world access, emphasizing provider shortages and underutilization.
- Muench A, Vargas I, Grandner MA, Ellis JG, Posner D, Bastien CH, et al. We know CBT-I works, now what? Fac Rev. 2022;11:4.10.12703/r/11-4.
  - This article synthesizes the field and emphasizes the need to extend CBT-I beyond efficacy toward broad public health impact.
- Harvey AG, Buysse DJ. Treating sleep problems: a transdiagnostic approach. New York: The Guilford Press; 2018.
  - This work introduces a transdiagnostic framework for treating sleep and circadian dysfunction, providing the conceptual foundation for mechanism-focused interventions.
- Harvey AG, Agnew ER, Esteva Hache R, Spencer JM, Diaz M, Ovalle Patino E, et al. A randomized trial of adapted versus standard versions of the Transdiagnostic Intervention for Sleep and Circadian Dysfunction implemented via facilitation and delivered by community mental health providers: improving the “fit” of psychological treatments by adapting to context. Implement Sci IS. 2025;20:32.10.1186/s13012-025-01440-9.
  - This study demonstrates that transdiagnostic sleep interventions can be effectively delivered by community providers with minimal training, supporting scalability and implementation.
- Sampson C, Bell E, Cole A, Miller CB, Marriott T, Williams M, et al. Digital cognitive behavioural therapy for insomnia and primary care costs in England: an interrupted time series analysis. BJGP Open. 2022;6:BJGPO.2021.0146. 10.3399/BJGPO.2021.0146.
  - This study demonstrates that digital CBT-I can reduce healthcare costs at scale.

## Data Availability

No datasets were generated or analysed during the current study.
